# Prevalence of Bacterial Species in Skin, Urine, Diarrheal Stool, and Respiratory Samples in Cats

**DOI:** 10.3390/pathogens11030324

**Published:** 2022-03-07

**Authors:** Dong Chan Moon, Ji-Hyun Choi, Naila Boby, Su-Jeong Kim, Hyun-Ju Song, Ho-Sung Park, Min-Chan Gil, Soon-Seek Yoon, Suk-Kyung Lim

**Affiliations:** 1 Bacterial Disease Division, Animal and Plant Quarantine Agency, 177 Hyeksin 8-ro, Gimcheon-si 39660, Gyeongsangbuk-do, Korea; ansehdcks@korea.kr (D.C.M.); wlgus01@korea.kr (J.-H.C.); nailaboby@korea.kr (N.B.); kimsujeong27@korea.kr (S.-J.K.); shj0211@korea.kr (H.-J.S.); medich777@naver.com (M.-C.G.); yoonss24@korea.kr (S.-S.Y.); 2 Division of Antimicrobial Resistance, National Institute of Health, Osong Health Technology Administration Complex, 187 Osong-eup, Cheongju 28159, Korea; 3 Department of Microbiology and Medical Science, Chungnam National University School of Medicine, Daejeon 35015, Korea; 89hos701@gmail.com

**Keywords:** prevalence, nationwide surveillance, companion animals, infections

## Abstract

Bacterial infections are a significant cause of illness and death in different animals. However, these bacterial infections could be a source of human disease or illness if these pathogenic bacteria are present in companion animals. This study aimed to investigate the prevalence of pathogenic bacteria associated with different site infections in cats in the Republic of Korea. For this purpose, samples were collected from the skin/ear, urine, respiratory, and diarrheal stool origins of cats obtained between 2018 and 2019 from seven different laboratories and centers participating in the Korean Veterinary Antimicrobial Resistance Monitoring System. These samples were subjected to analysis for the identification and isolation of associated bacterial species using a bacterial culture approach. A total of 609 isolates were identified in four different cat samples. Among them, 267, 184, 57, and 101 were extracted from diarrheal stool, skin, urine, and respiratory samples, respectively. The findings of this study showed that *Escherichia coli* was the most prevalent species among isolated bacterial species of diarrheal stool and urine origin. *Staphylococcus felis* and *Pasteurella multocida* were most prevalent in the skin and respiratory tract, respectively. However, there was no significant difference in bacterial distribution among the different age groups in all samples. This is the first nationwide surveillance report that associates bacterial prevalence with their site of origin and helps in the prevention of bacterial infections in cats. Moreover, the pattern of bacterial prevalence could provide sufficient guidance for the selection of empirical antimicrobial therapy against infections in cats.

## 1. Introduction

Currently, pet consumerism and pet-owning households are increasing due to structural changes in the population, mainly due to increased single-person households, changes in lifestyle, and aging [1]. In 2019, the proportion of South Korean pet-owning households was 5.91 households (26.4%), and an estimation showed that the pet industry will grow from 1544 to 3498 billion during 2018–2027 [2]. These growing factors have led to an increased interest in the health and wellbeing of companion animals to ensure a healthy and sound symbiotic companionship between animals and owners.

Furthermore, the emergence of bacterial infections in companion animals is another growing concern, as it affects global morbidity and mortality [3]. The sharing of a common environment between companion animals, especially dogs and cats, and their owners can be a possible factor for bacterial transmission. Friedmann et al. and Kim et al. reported that companion animals might play an integral role in transferring infections due to their direct contact with humans [1,4].

Bacterial prevalence studies are also increasing with the growing pet industry and bacterial infections. There have been limited studies on bacterial prevalence in companion animals within the Korean veterinary market. Thus, it is difficult to treat site-specific bacterial infections, and there is a possible increase in the extent of antimicrobial resistance among companion animal pathogenic bacteria in veterinary practice. In the present study, we aimed to report the nationwide prevalence of pathogenic bacteria in diarrheal stool, skin, urine, and respiratory tract infection specimens collected from cats at seven different laboratories/centers of metropolitan cities of Korea and participating in the Korean Veterinary Antimicrobial Resistance Monitoring System.

## 2. Materials and Methods

### 2.1. Research Design and Sample Analysis

We conducted a prevalence study for bacterial isolates collected and identified by laboratories and centers participating in the Korean Veterinary Antimicrobial Resistance Monitoring System from 2018 to 2019. In general, the isolates were collected in proportion to the number of veterinary hospitals in each city (Table 1). The clinical samples were obtained from the fecal, skin/ear, urine, and respiratory origins of cats, placed on ice, and transported to laboratories/centers participating in the monitoring system within 6 h of their collection.

### 2.2. Isolation and Identification of Bacteria

The swabs were directly plated on 5% defibrinated sheep blood agar (Hangang, Gunpo, South Korea) and MacConkey agar plates (MAC, BD, Spark, Baltimore, MD, USA) and incubated overnight at 37°C for 24 h under aerobic and anaerobic conditions. Only 1–2 major colonies showing morphological differences in one culture plate were selected for further analysis [5].

All the colonies were analyzed with matrix-associated laser desorption ionization time-of-flight mass spectrometry (microflex LT/SH spectrometer, bioMérieux, Germany) for identification. However, Staphylococcal species were identified by polymerase chain reaction using a previously reported method of identification [6].

### 2.3. Data and Statistical Analysis

Data were analyzed using Excel (Microsoft Office, USA) and GraphPad Prism 7 software (San Diego, CA, USA). Categorical variables were expressed as numbers and percentages. The threshold for statistical significance was set to a *p*-value of less than 0.05.

## 3. Results

### 3.1. Prevalence Pattern among Different Age Groups

A total of 609 bacteria were isolated from cats’ skin, diarrheal stool, urine, and respiratory samples during 2018–2019, nationwide from veterinary hospital-visited cats. The overall prevalence of bacteria was higher (45.2%) among cats aged 1–5 years, followed by 27.9% in cats from the 1-year age group. Bacterial isolates from diarrheal stool and skin samples were more frequently isolated than the other cat samples (Table 2). However, the prevalence pattern was different with variation in age groups, and most of the strains were isolated from cats aged less than 5 years. However, 23% of the bacteria were isolated from urine samples of 6–10-year age group cats.

### 3.2. Prevalence of Bacteria in Diarrheal Stool Samples

Overall, 267 isolates were collected and identified from diarrheal cat samples. Among the identified isolates, approximately 92.9% belonged to 13 different bacterial species. Among all identified bacteria from diarrheal stool samples, *Escherichia coli* was the most prevalent, with a prevalence of 65.5%. The prevalence of the identified bacteria was similar among the different regions. However, in cities where many bacteria are isolated, various strains were identified (Table 3).

**Figure 1:**
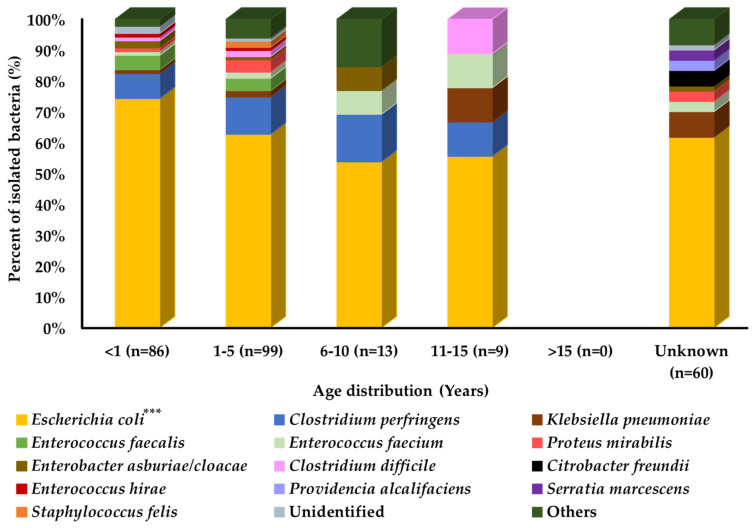
Bacterial prevalence (%) of isolates from cats’ diarrheal stool samples by age group (n = 267). In graph, “n” presents number of recovered isolates; * *p* < 0.05, ** *p* < 0.01, and *** *p* < 0.001.

Comparison of prevalence among different age groups of cats showed that the overall bacterial distribution pattern was similar in all age groups of cats, except for the older age group of cats. However, some bacterial species may differ in their prevalence among different age groups, such as *Klebsiella pneumoniae* and *Clostridium difficile*, which were most prevalent in cats aged 11–15 years, followed by the 1–5-year and <1-year age groups. *Enterococcus faecium* was most prevalent in cats aged 11–15 years, followed by the 6–10-year, 1–5-year, and <1-year age groups, as shown in Figure 1.

### 3.3. Prevalence of Bacteria in Skin Samples

Overall, 184 isolates from a variety of bacterial species (46 species) were collected and identified in cat skin samples. In this case, *Staphylococcus felis* (26.1%) was the most prevalent, followed by *Staphylococcus pseudintermedius* (12.5%) and *Staphylococcus schleiferi* (8.2%). However, seven bacterial species had a relatively low prevalence range of 2–5.5%. The identified bacterial species followed a similar pattern of prevalence in different cities (Table 4).

**Figure 2:**
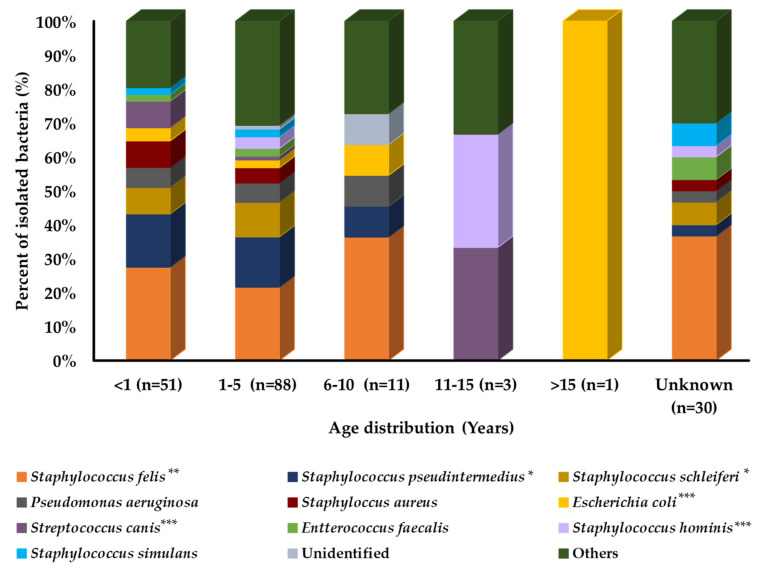
Bacterial prevalence (%) of isolates from cats’ skin samples by age group (n = 184). In graph, “n” presents number of recovered isolates; * *p* < 0.05, ** *p* < 0.01, and *** *p* < 0.001.

Prevalence analysis with age variation in cats showed that each bacterial species had a different distribution pattern among different age groups, as shown in Figure 2. *E. coli, Staphylococcus hominis,* and *Streptococcus canis* were the most prevalent and isolated bacteria from the old age group of cats. *Staphylococcus aureus, S. schleiferi*, and *Staphylococcus simulans* were the most prevalent in the younger age group of cats. However, *S. pseudintermedius* and *S. felis* were equally distributed among all age groups.

### 3.4. Prevalence of Bacteria in Urine Samples

Overall, 57 isolates were collected and identified in the urine samples of cats. Among the bacterial species isolated from urine samples, *E. coli* (17.5%) was the most prevalent, and *E. faecium* (8.8%) and *S. felis* (7%) were the second and third most prevalent species in urine samples from all cats, respectively. The overall prevalence of these three species was 33.3%, whereas the prevalence of other species ranged from 5.3% to 3.5%. However, it is difficult to compare the prevalence of individual species among different regions. The number of isolated strains was insufficient (Table 5).

**Figure 3:**
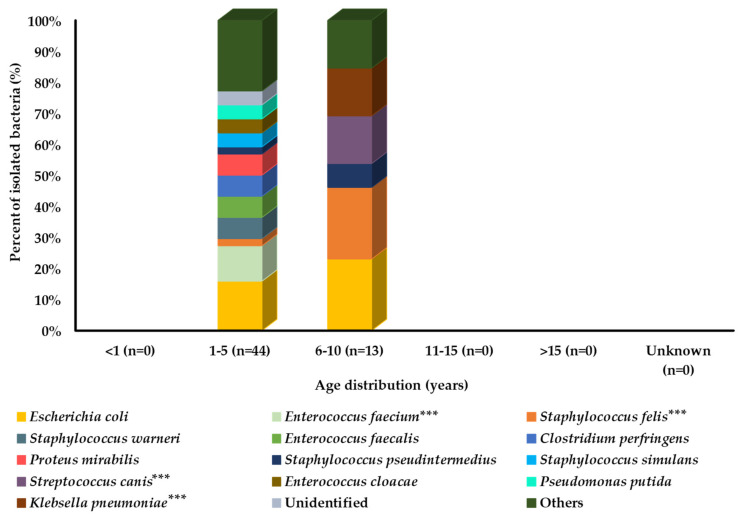
Bacterial prevalence (%) of isolates from cats’ urine samples by age group (n = 57). In graph, “n” presents number of recovered isolates; * *p* < 0.05, ** *p* < 0.01, and *** *p* < 0.001.

Among all age groups, bacteria were isolated only from the middle-aged groups (1–5 and 6–10 years). Among these two age groups, *E. coli* was the most prevalent strain. However, some bacterial species were more prevalent in cats aged 6–10 years compared with cats aged 1–5 years, including *S. felis*, *S. pseudintermedius*, *S. canis*, and *K. pneumoniae*. However, a large number of bacterial species showed a higher prevalence in the 1–5-year age group of cats, as shown in Figure 3.

### 3.5. Prevalence of Bacteria from the Respiratory Tract

Overall, 101 isolates were collected and identified from the respiratory tract of cats. A wide variety of bacterial species were identified in the respiratory specimens of cats. Different from other samples, *P. multocida* was the most prevalent (13.9%) bacterial species isolated from respiratory samples of cats, followed by *S. felis* (11.9%), *S. pseudintermedius* (9.9%), and *E. coli* (7.9%). However, the prevalence of other bacterial species was relatively lower in this case than in the other samples collected from the cats. Moreover, *Pasteurella dagmatis* (4%) and *Bordetella bronchiseptica* (3%) were two important respiratory tract pathogenic bacteria isolated from the respiratory specimens of cats (Table 6).

**Figure 4:**
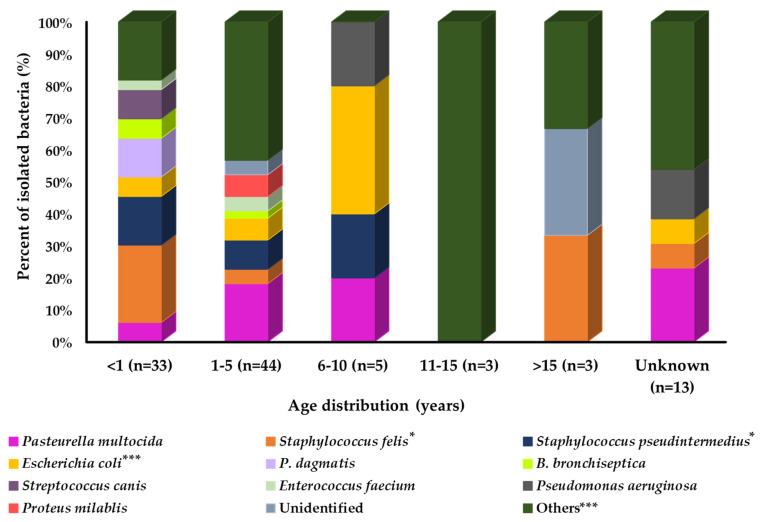
Bacterial prevalence (%) of isolates from cat’s respiratory samples by age group (n = 101). In graph, “n” presents number of recovered isolates; * *p* < 0.05, ** *p* < 0.01, and *** *p* < 0.001.

A prevalence study with age group variation showed that the prevalence of isolated bacteria was different among the groups (Figure 4). Among all age groups of cats, a large number of bacterial species were identified in the younger age group (<1 year). *P. dagmatics*, *B. bronchiseptica*, *S. canis,* and *E. faecium* were more prevalent in younger cats than in the other age groups. In contrast, *P. multocida* and *Proteus mirabilis* showed relatively higher prevalence in the middle and *S. felis* in the older age groups of cats. *Pseudomonas aeruginosa* and *E. coli* were more prevalent in the 6–10-year age group of cats. However, *S. pseudintermedius* was equally prevalent in the younger and middle-aged groups of cats compared with the older age group.

## 4. Discussion

Companion animals are important for their owners’ mental and physical health. However, the close contact between owners and companion animals may be a source of zoonotic infection transmission if these animals are carriers of virulent and resistant bacteria. Hence, prevalence monitoring studies are important to guide therapeutic decisions and the development of updated strategies to control infections.

In this report, we aimed to study the first nationwide prevalence of different bacteria isolated from different samples of cats in Korea. The distribution of bacterial species was dependent on the sampling site. However, among our isolated species, the most prevalent bacterial species were *E. coli* in diarrheal stool and urine samples, *S. felis* in skin samples, and *P. multocida* in respiratory tract samples of cats.

Moreover, the pattern of bacterial prevalence among cats with age variation was evaluated to elucidate the prevalence transition with age. The age-dependent prevalence transition study could assist veterinarians in identifying infectious and beneficial bacteria associated with cats from different age groups. According to the findings of our study, cats aged less than 5 years were more vulnerable to infections, with an increased prevalence of a variety of bacterial species compared with the other age groups.

As only a limited number of studies have reported previously on the bacterial prevalence in cats and age association, it is difficult to compare the present findings with other reports. However, our correlation of bacterial prevalence with age was in contrast to a prevalence study in house cats in Western Turkey. In this study, Muz et al. reported that there was no statistical difference observed between the prevalence of the pathogen in different age groups [7]. Furthermore, in our study, the most commonly isolated species of bacteria were *E. coli*, *S. felis*, *Enterococcus* spp., and *P. mirabilis* in cats, which agreed with the findings of Jung et al. in stray and hospital admitted cats of South Korea [8].

In the present study, our findings were consistent with those of a previous study by Polish researchers. Bierowiec et al. reported that *S. pseudintermedius* is the least prevalent bacteria in cats among the *Staphylococcus* class of bacteria [9]. In previous reports, the prevalence of *S. pseudintermedius* in dogs was 90%, which is higher than that in cats with a prevalence range of 2.49–8.8% [10,11,12,13,14,15]. Our results, together with those of previous reports, showed that *S. pseudintermedius* might not be a natural microbiota of cats.

Among diarrheal stool samples, *E. coli* was the most prevalent. Among the bacteria isolated from diarrheal stool samples, *Clostridium perfringens* was less prevalent than *E. coli.* The results of the present study are consistent with prior presumptions from North California that *C. perfringens* is of less clinical importance [16]. However, opposing results have also been reported in diarrheic cats by Oh et al. 2021. According to this study, *C. perfringens* was the predominant bacteria being isolated from the diarrheic cats [17]. These discrepancies could be related to varying factors, including regions, environmental conditions, methodologies, and concurrent diseases, thereby affecting the prevalence of bacterial species.

A comparison of the results obtained in this study showed a similar trend to that of international studies. Similar to the ComPath project of Europe [18], *S. felis* and *S. pseudintermedius* were the major isolates from skin samples, followed by *S. schleiferi* and *P. aeruginosa*.

Blanco et al. and Bartges et al. reported that bacterial urinary tract infections (UTIs) in cats are relatively rare events due to a variety of host defense mechanisms and that the reported prevalence varies, depending on the exclusion and inclusion criteria of the investigators [19,20]. Studies on cats with UTIs have reported that the overall bacterial prevalence of positive bacterial urine cultures is less than 3% [21,22,23].

In our study, we analyzed the relatively low bacterial prevalence in urine samples due to the small sample size compared with the other samples of cats. In this study, bacteria identified from UTIs can be expected to cause bacterial UTI in cats. *E. coli* is the most prevalent bacteria among the isolated bacteria, followed by *E. faecium* and *S. felis* in the urine samples of cats, echoing the results of other published reports in cats [20,24].

Moreover, *S. felis* was first recognized in feline clinical specimens and is considered a normal commensal organism present on the skin, conjunctival sac and eyelid margins, and saliva of normal healthy cats [25,26,27,28]. Litster et al. reported the strains of *S. felis* isolated from clinical specimens of cats with cystitis [24]. Consistent with these reports, we isolated *S. felis* as the most prevalent bacteria in skin samples of cats. In urine samples, it was the third most prevalent bacteria. The *Staphylococcal* species isolated from urine samples were *S. simulans*, *S. canis*, and *S. pseudintermedius*. Takahashi et al. in Japan reported their little pathogenic relativity with UTIs [29].

Furthermore, among the isolates collected from the respiratory tract samples of cats, *P. multocida* was the most prevalent, followed by *S. felis*, *S. pseudintermedius*, *E. coli*, *P. dagmatis*, *B. bronchiseptica*, *S. canis*, and *E. faecium.* In previous reports from the United Kingdom and the United States, Dolieslager et al. and Freshwater reported that *Pasteurella* species are not only part of the normal flora in cats but also an important zoonotic agent. According to them, subtypes of *Pasteurella* spp. have also been associated with human infections, including *P. multocida*, *P. canis*, *Pseudomonas septica*, *Pasteurella stomatis*, and *P. dagmatis* [30,31]. According to previous reports, *P. multocida* can be isolated from the pyothorax or subcutaneous abscesses and causative agents of secondary lower respiratory tract (LRT) infections in cats [32,33,34]. Other studies have reported that cat ownership by immunocompetent or immunocompromised persons carries the risk of *P. multocida* infections due to its transmission through cat bite, scratch, or respiratory secretions [35,36,37]. Based on our findings, some of the identified bacteria in respiratory samples were previously reported as common pathogenic bacteria in cats associated with LRT infections [38,39,40]. Different groups of researchers have reported that *B. bronchiseptica* is the primary pathogen in cats and in upper respiratory tract infections and is a co-pathogen with respiratory viruses/mycoplasma [41,42,43].

In our study, we isolated bacteria not only pathogenic to cats but also to humans. Overgaauw et al. reported that cat–owner companionship has significantly fewer positive health effects [44]. This study showed that the presence of zoonotic parasites and pathogens, including bacteria, viruses, and fungi, in healthy cats and dogs transmitted to humans through scratching, biting, licking, sneezing, and handling of pets consequently induced mild or severe bacterial infections. Another previous study reported that *P. multocida*, *Salmonella*, *S. canis*, *H. pylori*, *P. aeruginosa*, *K. pneumonia*, *Bordetella*, and *Corynebacterium bovis* are pathogenic bacteria associated with bacterial infections in humans and are transmitted through companion animals [44].

The companion animal population, especially cats, is likely to be more vulnerable to infectious diseases, leading to increased opportunities for antimicrobial administration. The emergence of antimicrobial resistance is another growing concern in veterinary practice. Thus, prevalence studies can guide the selection of appropriate treatments for each case and reduce bacterial resistance. Moreover, the findings of our study can create a paradigm for future studies on bacterial prevalence and antimicrobial susceptibility not only in cats but also in other companion animals, such as dogs.

## 5. Limitation and Future Perspectives

This study has some limitations such as the lack of information about the number of samples by region and season. Moreover, information about the sex, condition, and health status of cats was not provided, and several human-relevant bacteria have not been recovered in the study, such as EHEC (Enterohemorrhagic *E. coli*) and Salmonella, which might be due to a lack of specific culture media and growth conditions tested. However, prevalence data obtained from the present study could serve as a reference to start bacterial susceptibility and antimicrobial resistance monitoring in cats and be used to guide veterinarians’ decisions in clinical practices for the selection of antimicrobial agents to treat bacterial infections in cats throughout Korea. The findings of this study provide an improved understanding of bacterial prevalence for site-specific disorders among cats and can support veterinarians when listing differential diagnoses. Moreover, these findings can assist in the prioritization of therapeutics and health control strategies. Bacterial prevalence studies are not only important to attenuate the overuse of antimicrobials in veterinary practices but also help to reduce the spread of bacterial infections among companion animals and humans. There is a need to study the associated infections and susceptibility profiles of these bacteria in the future.

## Figures and Tables

**Table 1:** Total number of animal hospitals participated in this study from each city.

Cities	Number of Hospitals (%)		
	2018	2019	Total *
Seoul	10 (22.7)	15 (30.6)	18 (26.9)
Busan	5 (11.4)	9 (18.4)	10 (14.9)
Daegu	9 (20.5)	6 (12.2)	13 (19.4)
Incheon	4 (9.1)	7 (14.3)	7 (10.4)
Gwangju	3 (6.8)	3 (6.1)	5 (7.5)
Daejeon	5 (11.4)	5 (10.2)	5 (7.5)
Ulsan	8 (18.2)	4 (8.2)	9 (13.4)
Total	44 (100)	49 (100)	67 (100)

* Number of hospitals that appeared more than one time in the study period counted as one.

**Table 2:** Number of isolates from different samples and age groups of cats.

Samples	No. of Isolates (%)						
	<1	1–5	6–10	11–15	>15	Unknown	Total *
Diarrheal stool	86 (32.2)	99 (37.1)	13 (4.9)	9 (3.4)	-	60 (22.5)	267 (43.8)
Skin	51 (27.7)	88 (47.8)	11 (6)	3 (1.6)	1 (0.5)	30 (16.3)	184 (30.2)
Urine	-	44 (77.2)	13 (22.8)	-	-	-	57 (9.3)
Respiratory	33 (32.7)	44 (43.6)	5 (5)	3 (3)	3 (3)	13 (12.9)	101 (16.5)
Total **	170 (27.9)	275 (45.2)	42 (6.9)	15 (2.5)	4 (0.7)	103 (16.9)	609 (100)

* Total number of recovered isolates in each sample. ** Total number of recovered isolates in each age group.

**Table 3:** Most prevalent bacterial species in diarrheal stool samples of cats during 2018–2019.

Bacterial Species	No. of Isolates (%)							
	Seoul (n = 102)	Busan (n = 16)	Daegu (n = 10)	Incheon (n = 48)	Gwangju (n = 23)	Daejeon (n = 50)	Ulsan (n = 18)	Total (n = 267)
*Escherichia coli*	51 (50)	14 (87.5)	10 (100)	28 (58.3)	20 (87)	37 (74)	15(83.3)	175 (65.5)
*Clostridium perfringens*	13 (12.7)	-	-	-	1 (4.3)	7 (14)	1(5.6)	22 (8.2)
*Klebsiella pneumoniae*	3 (2.9)	1 (6.3)	-	5 (10.4)	-	-	-	9 (3.4)
*Enterococcus faecalis*	5 (4.9)	-	-	-	-	2 (4)	1(5.6)	8 (3)
*Enterococcus faecium*	5 (4.9)	-	-	-	1 (4.3)	-	1(5.6)	7 (2.6)
*Proteus mirabilis*	4 (3.9)	-	-	2 (4.2)	-	1 (2)	-	7 (2.6)
*Enterobacter cloacae*	3 (2.9)	-	-	1 (2.1)	-	1 (2)	-	5 (1.9)
*Clostridium difficile*	4 (3.9)	-	-	-	-	-	-	4 (1.5)
*Citrobacter freundii*	-	-	-	3 (6.3)	-	-	-	3 (1.1)
*Enterococcus hirae*	2 (2)	-	-	-	-	-	-	2 (0.7)
*Providencia alcalifaciens*	-	-	-	2 (4.2)	-	-	-	2 (0.7)
*Serratia marcescens*	-	-	-	2 (4.2)	-	-	-	2 (0.7)
*Staphylococcus felis*	2 (2)	-	-	-	-	-	-	2 (0.7)
Unidentified	3 (2.9)	-	-	-	1 (4.3)	-	-	4 (1.5)
Others *	7 (6.9)	1 (6.3)	-	5 (10.4)	-	2 (4)	-	15 (5.6)

* Others; 15 species.

**Table 4:** Most prevalent bacterial species in skin samples of cats during 2018–2019.

Bacterial Species	No. of Isolates (%)							
	Seoul (n = 58)	Busan (n = 22)	Daegu (n = 17)	Incheon (n = 26)	Gwangju (n = 18)	Daejeon (n = 29)	Ulsan (n = 14)	Total (n = 184)
*Staphylococcus felis*	14 (24.1)	5 (22.7)	2 (11.8)	4 (15.4)	10 (55.6)	9 (31)	4 (28.6)	48 (26.1)
*Staphylococcus pseudintermedius*	7 (12.1)	2 (9.1)	-	3 (11.5)	-	7 (24.1)	4 (28.6)	23 (12.5)
*Staphylococcus schleiferi*	5 (8.6)	2 (9.1)	1 (5.9)	4 (15.4)	-	3 (10.3)	-	15 (8.2)
*Pseudomonas aeruginosa*	3 (5.2)	4 (18.2)	-	1 (3.8)	-	2 (6.9)	-	10 (5.4)
*Staphylococcus aureus*	3 (5.2)	-	3 (17.6)	-	-	3 (10.3)	-	9 (4.9)
*Escherichia coli*	2 (3.4)	1 (4.5)	-	1 (3.8)	-	2 (6.9)	-	6 (3.3)
*Streptococcus canis*	-	2 (9.1)	3 (17.6)	-	1 (5.6)	-	-	6 (3.3)
*Enterococcus faecalis*	2 (3.4)	-	-	2 (7.7)	1 (5.6)	-	-	5 (2.7)
*Staphylococcus hominis*	1 (1.7)	-	2 (11.8)	1 (3.8)	1 (5.6)	-	-	5 (2.7)
*Staphylococcus simulans*	2 (3.4)	-	-	2 (7.7)	-	1 (3.4)	-	5 (2.7)
Unidentified	-	1 (4.5)	-	1 (3.8)	-	-	-	2 (1.1)
Others *	19 (32.8)	5 (22.7)	6 (35.3)	7 (26.9)	5 (27.8)	2 (6.9)	6 (42.9)	50 (27.2)

* Others; 36 species.

**Table 5:** Most prevalent bacterial species in urine samples of cats during 2018–2019.

Bacterial Species	No. of Isolates (%)						
	Seoul (n = 35)	Busan (n = 11)	Daegu (n = 1)	Incheon (n = 1)	Daejeon (n = 3)	Ulsan (n = 6)	Total (n = 57)
*Escherichia coli*	8 (22.9)	-	-	1 (100)	1 (33.3)	-	10 (17.5)
*Enterococcus faecium*	5 (14.3)	-	-	-	-	-	5 (8.8)
*Staphylococcus felis*	2 (5.7)	1 (9.1)	-	-	1 (33.3)	-	4 (7)
*Staphylococcus warneri*	-	2 (18.2)	-	-	-	1 (16.7)	3 (5.3)
*Enterococcus faecalis*	2 (5.7)	1 (9.1)	-	-	-	-	3 (5.3)
*Clostridium perfringens*	3 (8.6)	-	-	-	-	-	3 (5.3)
*Proteus mirabilis*	1 (2.9)	2 (18.2)	-	-	-	-	3 (5.3)
*Staphylococcus pseudintermedius*	-	-	-	-	1 (33.3)	1 (16.7)	2 (3.5)
*Staphylococcus simulans*	2 (5.7)	-	-	-	-	-	2 (3.5)
*Streptococcus canis*	-	2 (18.2)	-	-	-	-	2 (3.5)
*Enterobacter cloacae*	2 (5.7)	-	-	-	-	-	2 (3.5)
*Pseudomonas putida*	2 (5.7)	-	-	-	-	-	2 (3.5)
*Klebsiella pneumoniae*	1 (2.9)	-	-	-	-	1 (16.7)	2 (3.5)
Unidentified	2 (5.7)	-	-	-	-	-	2 (3.5)
Others *	5 (14.3)	3 (27.3)	1 (100)	-	-	3 (50)	12 (21.1)

* Others; 12 species.

**Table 6:** Most prevalent bacterial species in respiratory samples of cats during 2018–2019.

Bacterial Species	No. of Isolates (%)							
	Seoul (n = 38)	Busan (n = 2)	Daegu (n = 3)	Incheon (n = 27)	Gwangju (n = 10)	Daejeon (n = 5)	Ulsan (n = 16)	Total (n = 101)
*Pasteurella multocida*	9 (23.7)	-	-	1 (3.7)	3 (30)	-	1 (6.3)	14 (13.9)
*Staphylococcus felis*	-	-	-	6 (22.2)	1 (10)	2 (40)	3 (18.8)	12 (11.9)
*Staphylococcus pseudintermedius*	3 (7.9)	-	-	6 (22.2)	-	-	-	10 (9.9)
*Escherichia coli*	3 (7.9)	-	-	1 (3.7)	-	2 (40)	2 (12.5)	8 (7.9)
*Pasteurella dagmatis*	-	1 (50)	-	2 (7.4)	-	-	1 (6.3)	4 (4)
*B. bronchiseptica*	-	1 (50)	-	2 (7.4)	-	-	-	3 (3)
*Streptococcus canis*	-	-	-	2 (7.4)	-	-	1 (6.3)	3 (3)
*Enterococcus faecium*	2 (5.3)	-	-	-	-	-	1 (6.3)	3 (3)
*Pseudomonas aeruginosa*	-	-	-	1 (3.7)	-	-	-	3 (3)
*Proteus mirabilus*	2 (5.3)	-	-	1 (3.7)	-	-	-	3 (3)
Unidentified	1 (2.6)	-	-	1 (3.7)	-	-	1 (6.3)	3 (3)
Others *	18 (47.4)	-	-	4 (14.8)	6 (60)	1 (20)	6 (37.5)	35 (34.7)

* Others; 27 species.

## Data Availability

The data that support the findings of this study are available from the corresponding author upon reasonable request.
